# Robot-assisted minimally invasive photoacoustic imaging for monitoring liver ablation using diffusing fiber illumination

**DOI:** 10.1117/1.JBO.31.12.123302

**Published:** 2026-02-20

**Authors:** Shang Gao, Xihan Ma, Yanbo Hua, Sharath Bhagavatula, Guigen Liu, Oliver Jonas, Haichong K. Zhang

**Affiliations:** a Worcester Polytechnic Institute, Department of Robotics Engineering, Worcester, Massachusetts, United States; b Massachusetts General Hospital, Harvard Medical School, Wellman Center for Photomedicine, Boston, Massachusetts, United States; c Massachusetts General Hospital, Harvard Medical School, Cardiovascular Research Center, Cardiology Division, Boston, Massachusetts, United States; d Brigham and Women’s Hospital, Harvard Medical School, Department of Radiology, Boston, Massachusetts, United States; e Worcester Polytechnic Institute, Department of Biomedical Engineering, Worcester, Massachusetts, United States; f Worcester Polytechnic Institute, Department of Computer Science, Worcester, Massachusetts, United States

**Keywords:** photoacoustic imaging, robot, spectral analysis, diffusing fiber, minimally invasive, liver ablation

## Abstract

**Significance:**

Accurate intraoperative assessment of ablation completeness in liver radiofrequency ablation (RFA) remains a clinical challenge, as conventional imaging lacks real-time capability to delineate necrotic boundaries. Incomplete ablation increases recurrence risk, underscoring the need for real-time, high-resolution imaging with functional tissue differentiation.

**Aim:**

We propose a robot-assisted photoacoustic (PA) imaging system employing a customized diffusing optical fiber to improve intraoperative monitoring of liver RFA.

**Approach:**

The system integrates circumferential wide-field illumination for enhanced tissue coverage with robotic automated 3D scanning and co-registered ultrasound. Spectroscopic PA imaging differentiates necrotic from viable tissue based on optical absorption, whereas a standard Hough transform algorithm suppresses fiber-induced artifacts. Validation was performed using *ex vivo* and cadaveric swine liver studies.

**Results:**

In cadaveric studies, 3D lesion mapping showed necrotic zones of 7.55×5.37×7.42  mm, closely matching gross pathology measurements (7.93 mm average diameter), confirming system accuracy.

**Conclusions:**

The proposed system enables accurate, real-time visualization of ablation lesions *in situ*, offering a clinically viable approach to improve treatment precision and reduce recurrence in liver RFA procedures.

## 1 Introduction

Liver cancer, including hepatocellular carcinoma (HCC) and colorectal liver metastases (CLM), remains a significant contributor to global cancer mortality. HCC is the sixth most common cancer worldwide and the third leading cause of cancer-related deaths.^1^ CLM occurs frequently in patients with advanced colorectal cancer, with the liver being the most common site for metastatic spread due to its dual blood supply and filtration role.^2^ These tumors are frequently diagnosed at a stage when surgical resection is required.^3^ However, tumors are considered unresectable under certain conditions, such as prohibitive comorbidities, unfavorable tumor location, or advanced liver disease.^4^^,^^5^

Radiofrequency ablation (RFA) has emerged as a minimally invasive alternative for patients with unresectable liver tumors. It involves inserting a needle electrode into the tumor under imaging guidance and delivering high frequency alternating current to generate localized heat and induce coagulative necrosis. Despite its clinical advantages, including reduced hospital stay, repeatability, and suitability for patients unfit for surgery,^6^^–^^8^ RFA has a relatively high rate of local tumor recurrence. This issue is often caused by incomplete ablation, especially in deep or irregularly shaped lesions. It largely stems from the inability to visualize ablation-induced necrosis with sufficient clarity during the procedure.^9^^–^^11^

Conventional imaging techniques used in current clinical practice to guide liver RFA fall short of visualizing the ablation boundary. Ultrasound (US) is widely used for needle guidance and tumor localization in real time. But conventional US lacks the contrast sensitivity to distinguish necrotic from viable tissue. Although contrast-enhanced US (CEUS) is capable of detecting necrosis, its effects are transient and do not support real-time monitoring.^12^ Computed tomography (CT) and magnetic resonance imaging (MRI) can assess necrosis. Yet, their low imaging framerate makes it difficult for intraoperative guidance. Also, MRI is not accessible in many places due to cost and limited availability. Photoacoustic (PA) imaging, on the other hand, offers a hybrid solution that combines the molecular contrast of optical imaging with the resolution of ultrasound. In PA imaging, short laser pulses excite chromophores within the tissue, such as hemoglobin or water, generating ultrasonic waves due to thermoelastic expansion. The detected acoustic waves are then reconstructed into images that reflect the tissue’s optical absorption properties.^13^ Spectroscopic PA (sPA) further enhances this approach by using multiple wavelengths to capture tissue-specific absorption spectra, thereby enabling functional differentiation between healthy and ablated tissue based on their distinct spectral signatures.^14^^–^^16^

Several studies have demonstrated the feasibility of sPA imaging in detecting RFA lesions based on PA spectral difference.^17^^–^^21^ PA-based ablation guidance has been extensively investigated in cardiac applications, with accurate boundary detection and *in vivo* demonstration to compensate for motion artifact on beating heart.^22^^,^^23^ The same approach has been demonstrated with *ex vivo* hepatic tissues.^21^ However, a major challenge remains in the effective delivery of light. Most PA systems use surface-illumination schemes with fiber bundles or single-tip optical fibers that provide limited penetration and poor volumetric coverage, especially in deep organs such as the liver. In interventional settings, there is a pressing need for miniaturized, flexible, and highly efficient light delivery systems that can be deployed with minimal disruption to the surgical workflow. Although tools have been developed to deploy PA imaging for minimally invasive cardiac ablation,^24^ in contrast to liver RFA, light can be delivered directly to the heart surface via chamber illumination, enabling a wider field of view. The biopsy-style approach used in liver RFA presents additional challenges for implementing PA imaging in ablation monitoring. Kruit et al.^25^ proposed an annular fiber probe to improve interstitial light delivery and needle visibility in liver RFA guidance. Yet, this approach lacked spectroscopic contrast for tissue assessment. Bhagavatula et al.^26^ developed a needle-integrated PA system enabling real-time US and PA imaging with submillimeter resolution. But the system used single-wavelength illumination and did not demonstrate tissue characterization. In addition, these approaches require the US transducer to receive signals from the tissue surface, which introduces challenges in acoustic-optic alignment, particularly when imaging targets deep within the organ.

**Fig. 1:**
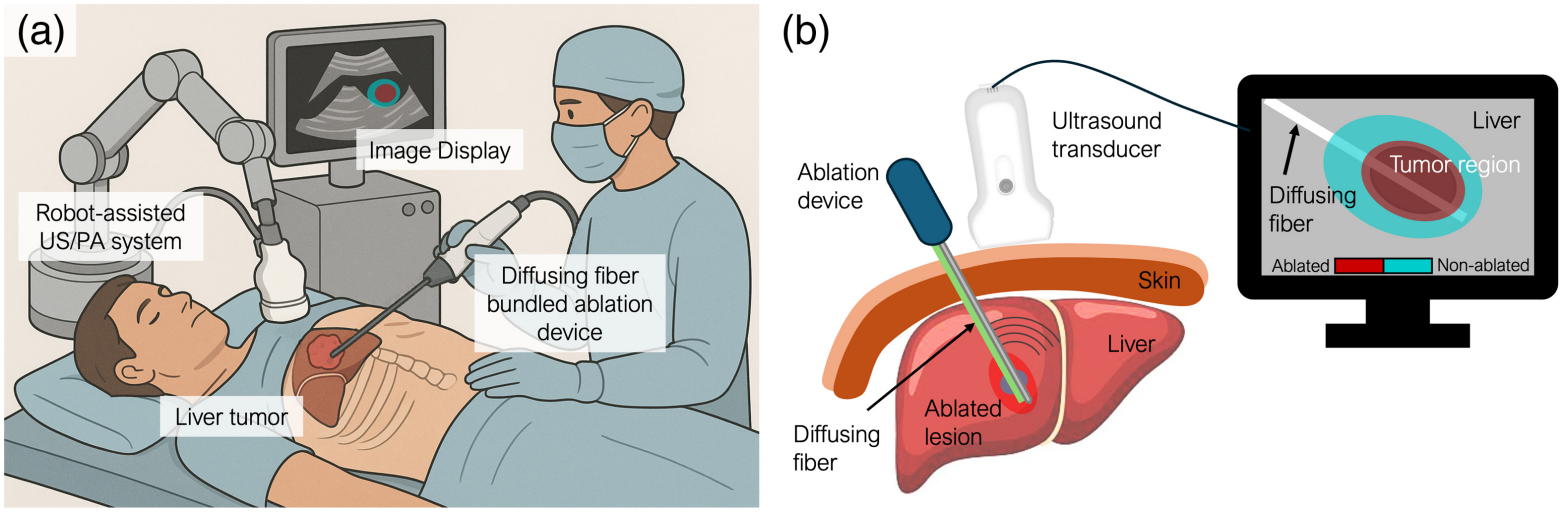
Robot-assisted minimally invasive photoacoustic (PA) imaging for monitoring liver ablation. (a) Illustration of a robotic ultrasound (US)/PA system used to image the liver tumor and ablation boundary intraoperatively while a clinician operates a diffusing fiber bundled ablation device. (b) Schematic showing the insertion of the diffusing fiber into the liver, enabling precise localization and visualization of the tumor and the ablation boundary.

To address this challenge, we introduce a novel robot-assisted minimally invasive PA imaging system using a diffusing optical fiber approach that emits light circumferentially along a treated segment of its shaft. This side-illumination design enables broader and more uniform PA excitation within the tissue volume, reducing the need for multiple insertions or repositioning of the fiber. The diffusing fiber is fabricated using a simple chemical etching technique and is compatible with clinical ablation tools and sterile procedures. Furthermore, our study advances intraoperative imaging by integrating the PA system into a robotically controlled platform capable of precise and repeatable three-dimensional (3D) scanning.^27^^–^^31^ The robotic arm, equipped with a linear-array ultrasound probe, performs automated scanning over the liver area on the patient’s body surface, acquiring co-registered volumetric ultrasound and PA datasets. This 3D approach is crucial for assessing lesion shape, size, and margins, which conventional 2D imaging often fails to capture. The concept of our proposed framework is illustrated in Fig. 1. We validate the proposed method and workflow in a swine cadaver model, showcasing its feasibility for intraoperative liver RFA assessment. By combining broad-field illumination from the diffusing fiber with high-resolution 3D robotic scanning, we offer a powerful tool for intraoperative assessment of ablation-induced lesion in liver RFA procedure.

The contribution of this paper lies in three key aspects: (1) We propose the use of a diffusing optical fiber for photoacoustic illumination during liver biopsy, enabling wide-field excitation from a single-site insertion. (2) We integrate a robot-assisted platform into the PA-guided workflow to improve imaging alignment and enable 3D data acquisition. (3) We demonstrate the feasibility of a robot-assisted, PA-guided liver RFA procedure *in situ* on a swine cadaver model, showcasing its clinical applicability. The paper is structured as follows: We first detail the architecture of the proposed robotic imaging system and the algorithm for identifying ablation-induced lesions using PA imaging. Subsequently, we describe the experimental setup, *ex vivo* evaluation, and preparation for the animal cadaver demonstration. Our results validate the feasibility of using a diffusing fiber for PA imaging to guide liver RFA procedures and establish the functionality of the robotic PA imaging workflow. Finally, we discuss the study’s findings and limitations. Preliminary results from this work have been previously presented in conference proceedings.^32^^,^^33^

## 2 Methods

### 2.1 Robot-Assisted Minimally Invasive Photoacoustic Imaging System

**Fig. 2:**
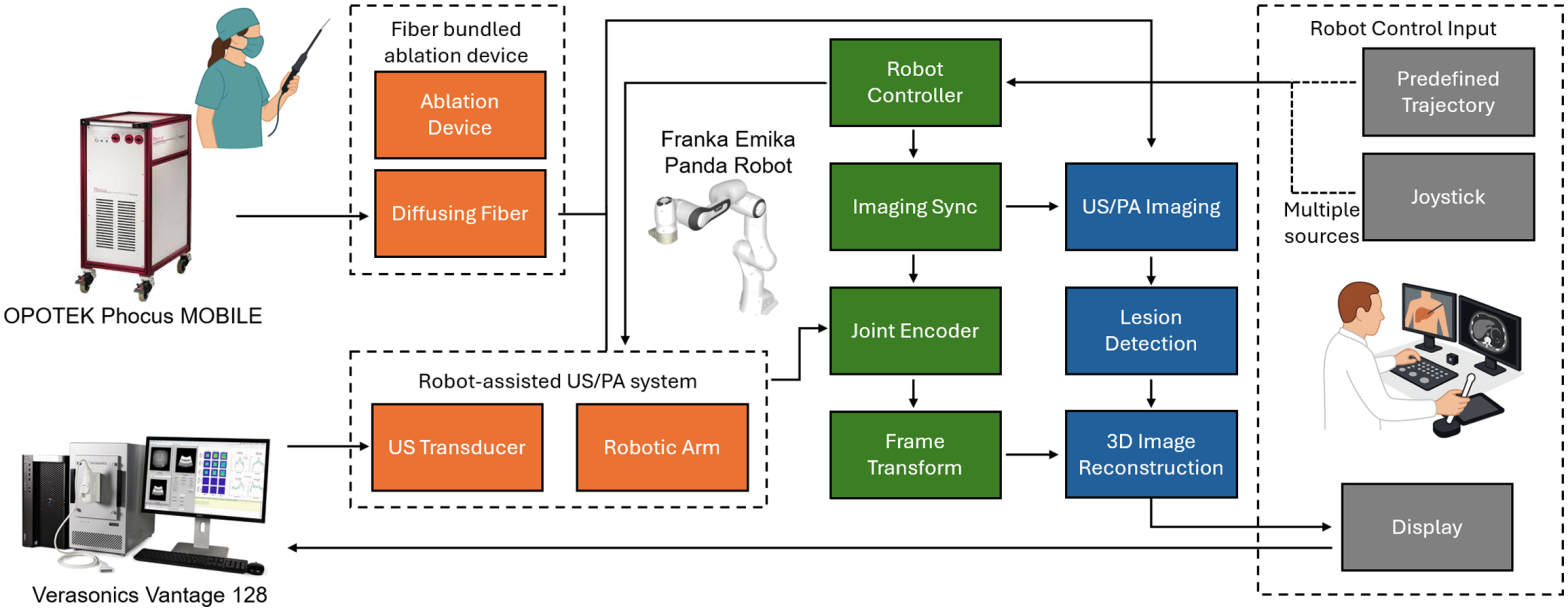
System architecture and communication framework of the proposed system.

A robot-assisted wide-area PA imaging system was developed to enable large field-of-view intraoperative imaging, particularly for liver applications.^34^ An overview of system architecture and communication framework is shown in Fig. 2. The system utilized a 7-degree-of-freedom robotic arm (FR3, Franka Emika, Germany) to actuate a linear US transducer (L12-5 50 mm, Philips, Netherlands). The transducer has a center frequency of 7.5 MHz with a bandwidth between 5 and 12 MHz. The transducer was connected to a US data acquisition system (Vantage 128, Verasonics, USA).

The robotic platform supported both teleoperated control and predefined trajectory execution. During operation, the controller maintains constant probe-tissue contact pressure and orientation to ensure consistent acoustic coupling and imaging quality. For this study, the operator manually positioned the probe at the starting location, after which a predefined raster trajectory with 2 mm step intervals was executed. The robot advanced incrementally along the y-axis (imaging elevational axis) while autonomously adjusting its z-axis (imaging axial) position to maintain surface contact, minimizing probe tilt and lateral drift.

The diffusing fibers used in the probe were fabricated by chemically etching the sidewalls of multimode optical fibers (FT600EMT, Thorlabs, USA), enabling lateral emission for broader light distribution. This fabrication method enhanced the excitation area and homogenized illumination across the imaging field, improving lesion boundary visibility during PA scanning.^35^ The laser source (Phocus MOBILE, OPOTEK, USA) provided wavelength-tunable output (690–950 nm, 20 Hz, and 5 ns pulse duration) for spectroscopic PA imaging. The laser system was controlled by the US system.

Synchronization between the robotic actuation and imaging system was managed via a bilateral communication interface built with the Robot Operating System (ROS). The robot followed a move–stop–scan sequence, pausing at each predefined position for synchronized PA/US data acquisition. Motion and imaging triggers were coordinated through the ROS interface to ensure precise temporal alignment. Each 2D PA image was associated with a corresponding robot pose, enabling accurate registration of all frames into a unified global coordinate system for volumetric reconstruction.

### 2.2 Photoacoustic Imaging for Ablation Monitoring

sPA imaging was employed to differentiate ablated tissue from surrounding nonablated tissue based on spectral characteristics. Nonablated tissue typically exhibited spectra resembling deoxygenated hemoglobin (HbR), with a local spectral peak near 760 nm absent in ablated tissue. Multiple wavelengths (typically 700 to 850 nm) were used to excite the tissue and capture tissue-specific spectral signatures.^17^^,^^21^ Spectral decomposition was applied under the assumption that the measured PA signal is a linear combination of absorption contributions from ablated and nonablated tissue. The decomposition yielded two basis spectra representing each tissue state, and the relative contribution from each was estimated using a least-squares optimization approach.^22^

The ablation index was then computed as the ratio of ablated tissue intensity to the total tissue signal, providing a continuous measure of ablation progression rather than a binary classification. A residual spectral filter was applied to remove pixels with insufficient illumination or excessive noise, ensuring that only spectrally valid pixels were used for analysis. Finally, a threshold-based segmentation was applied to the computed ablation index map to visualize the ablation-induced lesion boundary.^23^ This approach, validated in previous studies, provides robust, quantitative feedback on ablation extent and spatial distribution of necrosis.

### 2.3 Standard Hough Transformation Masking

**Fig. 3:**
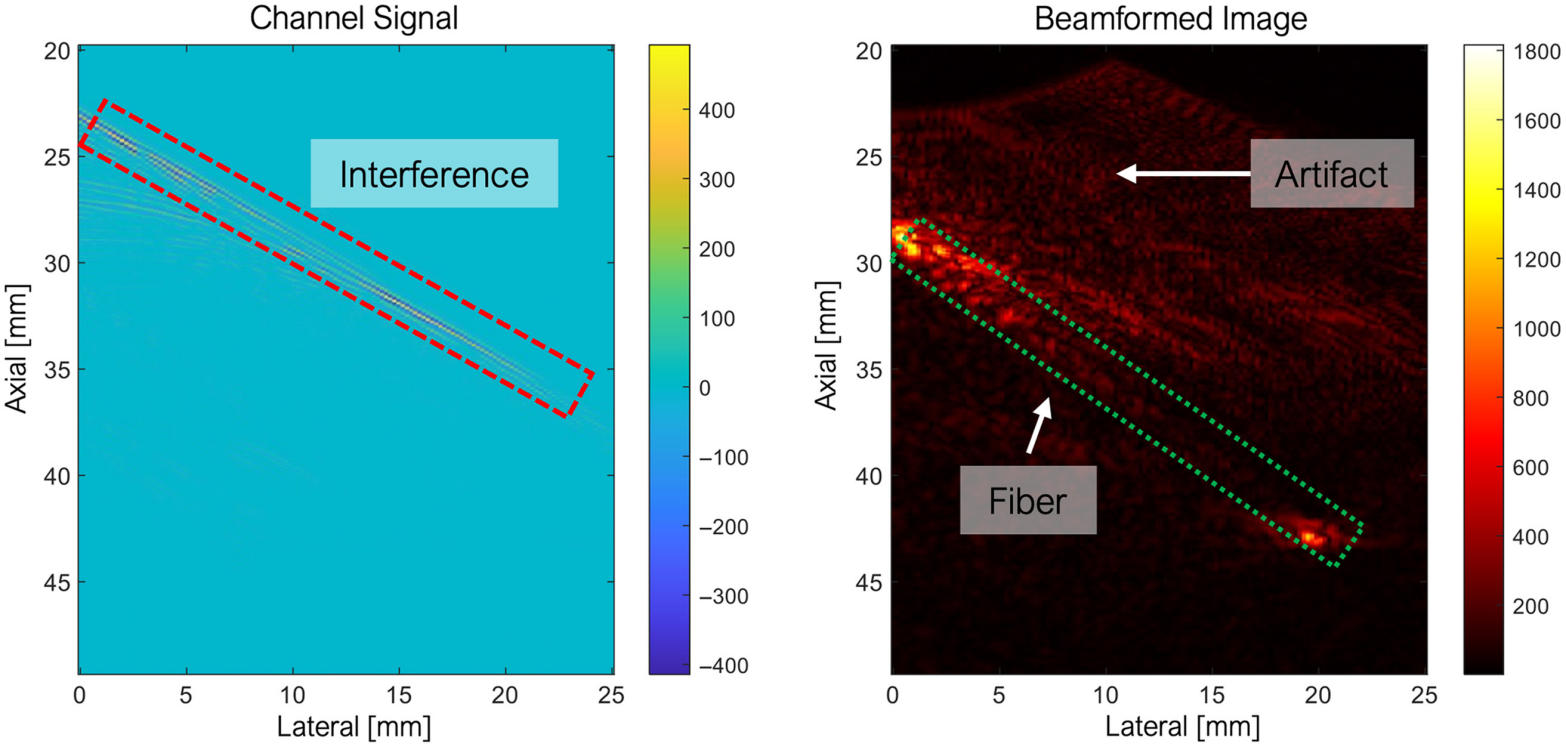
Fiber-induced signal artifacts: high-intensity photoacoustic (PA) signal interference was observed in the channel signal along the diffusing fiber.

Fiber-induced PA signal artifacts were addressed using a standard Hough transform (SHT) masking algorithm. Signal artifacts caused by sidelobe interference along the axis of the diffusing fiber were detected as line features in the raw channel data, as shown in Fig. 3. SHT was used to identify these linear features, and a binary mask was applied prior to beamforming to suppress artifacts. This preprocessing step improved contrast and accuracy in mapping ablated lesions and minimized false-positive necrotic signals.

### 2.4 Volumetric Ultrasound/Photoacoustic Imaging

To enable the visualization of ablation area in the liver region, a sweeping motion of the US/PA probe is implemented to collect volumetric data across the tissue over the liver area. The probe follows either a teleoperation or a predefined trajectory. During scanning, the probe periodically pauses at fixed spatial intervals to acquire multiwavelength PA images, then resumes motion along the path.^34^^,^^36^

At each acquisition point, the spatial relationship between the robot base frame $\left\{F_{\text{base}}\right\}$ and the probe frame $\left\{F_{\mathrm{PA}}\right\}$ is captured as a rigid transformation $T_{i}\in SE(3)$. Upon completing the scan, a total of N probe poses and N corresponding 2D PA images are obtained.

To construct a 3D PA volume, each 2D image is transformed into the common coordinate frame, $\left\{F_{\text{base}}\right\}$, using the recorded transformations. For a pixel $P_{i}^{\mathrm{PA}}$ in the i’th PA image, defined in the probe’s frame, its spatial location in the robot base frame, $P_{i}^{\text{base}}$, is computed by Eq. (1).

$$\lfloor P_{i}^{\text{base}}\rfloor =T_{i}P_{i}^{\mathrm{PA}}.$$ (1)

Here, the coordinates are expressed in homogeneous form. The resulting 3D dataset is generated by discretizing these transformed pixel positions into voxel space.

## 3 Experimental Implementation and Animal Preparation

### 3.1 Diffusing Fiber Illumination Field

A custom-fabricated optical diffusing fiber was developed to achieve uniform and wide-field illumination within liver tissue. The illumination performance of the diffusing fiber was assessed in *ex vivo* liver experiments by comparing it to a conventional tip-illumination fiber. To evaluate spatial light distribution, 33 steel needles were embedded in the liver at 2.5 mm intervals using a 3D-printed positioning fixture. PA images were acquired using both the diffusing and tip-illumination fibers. The PA signal intensity at each needle location served as a quantitative measure of the excitation fluence profile for both illumination strategies.

### 3.2 *Ex Vivo* Ablation-Induced Lesion Detection

The utility of the diffusing fiber for detecting ablation-induced necrosis was further demonstrated in a separate *ex vivo* porcine liver study. Tissue samples, sourced from a local market, were ablated using an irrigated RFA catheter (Safire BLU Duo SP, St. Jude Medical, USA) at 35 W with a 17  mL/min irrigation rate. The same US/PA system was used to image the tissue following ablation. Sixteen wavelengths ranging from 700 to 850 nm were selected for sPA imaging to capture the wavelength-dependent optical absorption characteristics of the tissue. This wavelength range was selected based on the known spectral features of previously reported ablation-induced spectral changes.^17^^,^^21^ In particular, a local spectral peak near 760 nm has been shown to weaken or disappear after ablation. By covering this range with 10 nm intervals, the system enabled accurate differentiation between ablated and nonablated regions through analysis of their spectral signatures. This approach allowed robust identification of necrotic tissue regions and validation of the diffusing fiber’s capability for uniform and efficient PA illumination.

### 3.3 *In Situ* Cadaver Ablation and Scanning

**Fig. 4:**
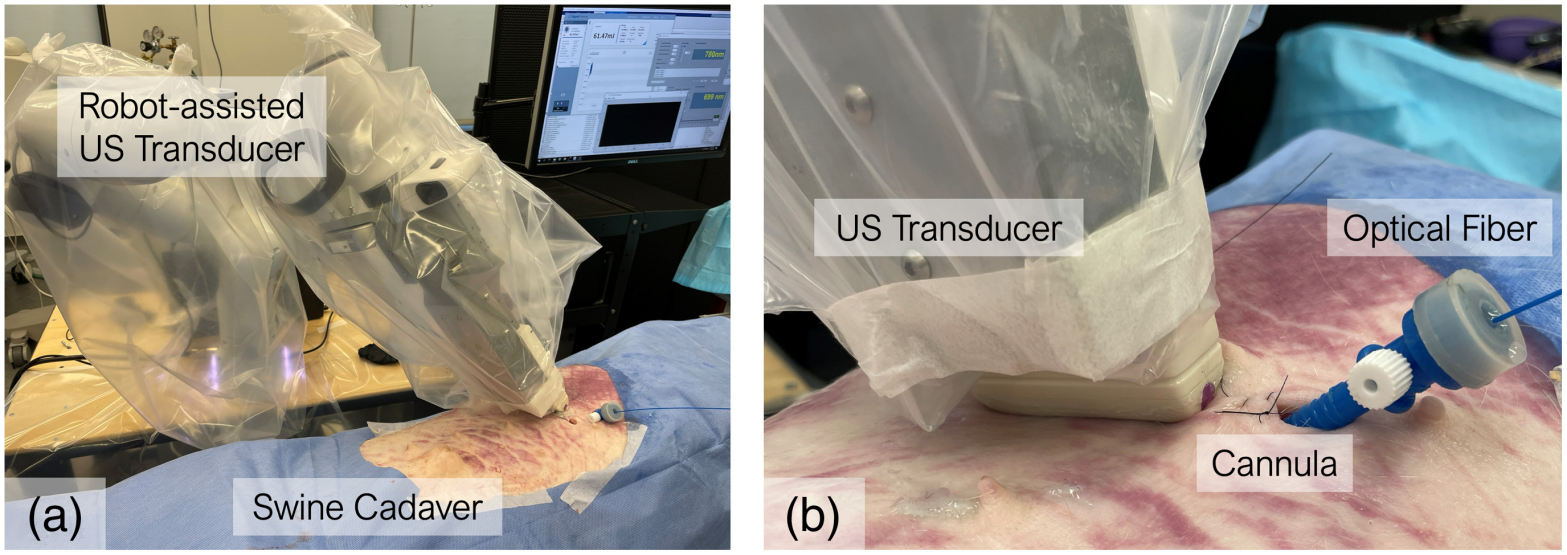
Cadaver study setup: (a) a robot-assisted ultrasound (US) transducer was used to receive US/photoacoustic (PA) signals from the skin surface. (b) The ablation device and optical fiber were inserted into the liver through a cannula.

The effectiveness of the diffusing fiber for detecting ablated liver tissue, along with artifact suppression, was evaluated *in situ* using a swine cadaver model (male Yorkshire, 50.7 kg). The experiment was performed two days post-euthanasia. The cadaver was shaved and positioned in a supine orientation to allow clear access to the liver, as illustrated in Fig. 4. A surgical incision was made, and a cannula was secured to guide fiber insertion into the liver. Using US guidance, the diffusing fiber was introduced through the cannula. Prior to ablation, PA and US imaging were conducted using a wavelength sweep from 700 to 850 nm with 10 nm increments, capturing 64 frames per wavelength to improve image contrast.

Subsequently, RFA was performed on the liver using an irrigated ablation catheter (Safire BLU Duo SP, St. Jude Medical, USA) inserted under US guidance. The ablation was carried out at 35 W power with a 17  mL/min irrigation rate. After the procedure, the diffusing fiber was reinserted into the vicinity of the ablated region based on US localization, and a second round of US/PA imaging was conducted using the same acquisition parameters to visualize and localize the necrotic zone.

To further capture the spatial extent of the lesion, robot-assisted 3D tomography was performed along the skin surface over the liver area. A total of 20 cross-sectional PA frames were acquired in a trajectory parallel to the inserted diffusing fiber. To increase scanning speed during wide area scanning, fewer wavelengths were used for sPA imaging. Five wavelengths (710, 740, 760, 780, and 850 nm) were selected for spectroscopic imaging, with 10-frame averaging at each step to improve signal quality. These wavelengths capture the primary spectral differences between ablated and nonablated tissue, emphasizing the local signal peak near 760 nm. These multispectral volumetric scans enabled reconstruction of the 3D lesion boundary, offering a comprehensive view of the ablated region within the liver tissue.

A gross pathological comparison was conducted between the post-scan tissue sample and the necrotic area identified by PA to assess detection accuracy. The liver tissue was stained using a 2% solution of 2,3,5-triphenyltetrazolium chloride (TTC) in 0.9% saline for 20 min at 37°C to improve visualization of the ablation lesion.^37^^,^^38^ TTC serves as a marker of metabolic activity and is widely recognized for identifying tissue damage in experimental models. This colorless, water-soluble dye is reduced by the mitochondrial enzyme succinate dehydrogenase in viable cells to produce formazan, a light-sensitive, water-insoluble compound that stains healthy (nonablated) tissue a deep red. By contrast, damaged (ablated) regions, lacking metabolic activity, do not undergo this reaction and thus remain white, clearly indicating tissue injury.

## 4 Results

### 4.1 Diffusing Fiber Illumination Field

**Fig. 5:**
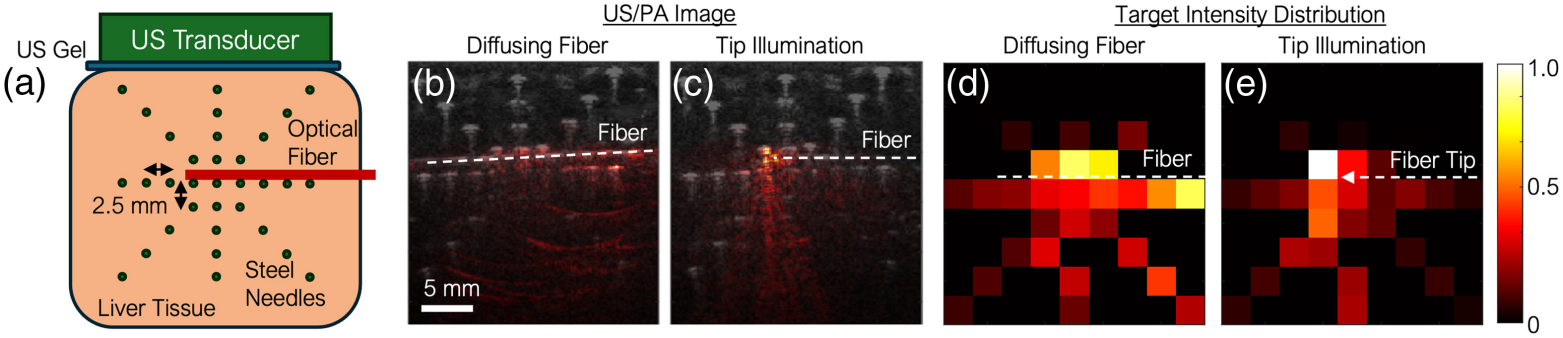
Photoacoustic (PA) excitation area comparison between diffusing and tip illumination. (a) Experiment setup sketch. (b), (c) Fiber illumination US/PA images. (d), (e) Intensity distribution across needle targets displayed using the same colormap and dynamic range to enable direct comparison between the two illumination methods.

We assessed the diffusing fiber’s effectiveness for ablation monitoring through *ex vivo* experiments. The phantom setup was shown in Fig. 5(a). Illumination coverage between the diffusing fiber and a conventional tip-illumination fiber was compared. PA images of steel needles embedded in liver tissue were co-registered with corresponding US images. As shown in Fig. 5(b), the diffusing fiber provided broader illumination, whereas Fig. 5(c) illustrates the limited coverage of the tip illumination. For quantitative evaluation, each pixel in the PA map corresponds to a single needle tip, and its intensity represents the mean absolute PA signal within a fixed-size region centered on that tip. Figures 5(d) and 5(e) present the normalized intensity maps. A common colormap and dynamic range were applied for both images to enable direct visual comparison. The normalized intensity maps demonstrate that the diffusing fiber produces a more uniform illumination profile across a wider area compared with the localized energy delivery of the tip-illumination fiber.

### 4.2 *Ex Vivo* Ablation-Induced Lesion Detection

**Fig. 6:**
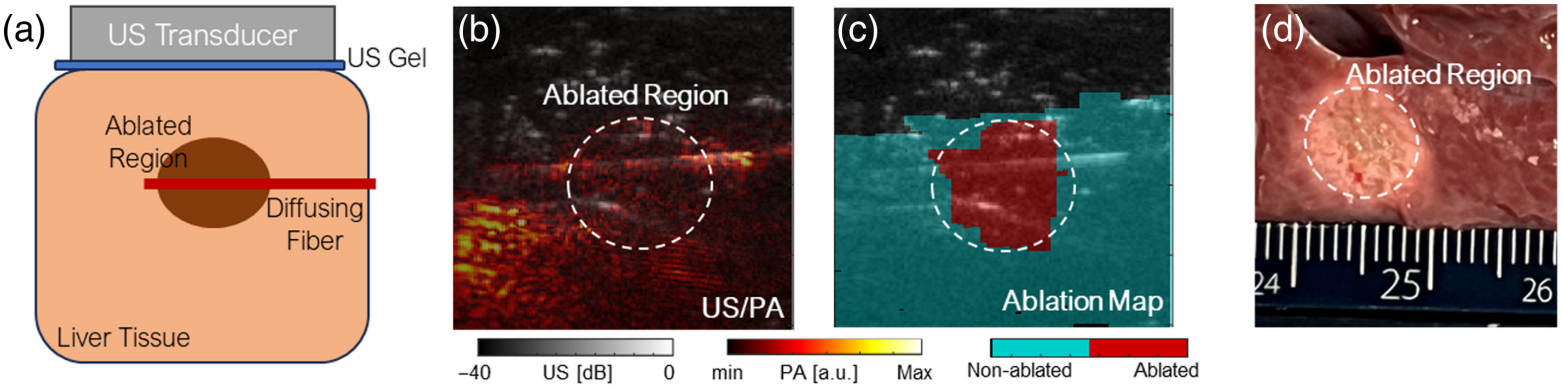
*Ex vivo* liver ablation study using diffusing fiber illumination. (a) Experiment setup. (b) Photoacoustic (PA) image of ablated liver tissue at 700 nm wavelength. (c) PA-based lesion map highlights the ablated lesion. (d) Gross pathology measurement of lesion size post-ablation.

The use of the diffusing fiber to identify ablation-induced lesions in liver tissue was further validated, as illustrated in Fig. 6(a). The fiber was laterally positioned to intersect the necrotic region. It should be noted that the ablated region is not consistently distinguishable in single-wavelength PA images, as the intensity contrast between ablated and nonablated tissue is often minimal. Therefore, differentiation relies on multiwavelength spectroscopic analysis. Spectrally unmixed sPA images [Fig. 6(b)] were generated using reference spectra for ablated and nonablated tissues. An ablation index was calculated to delineate the lesion, and the resulting boundary was compared with gross pathology measurements obtained after the procedure. Three liver tissue samples were ablated and subsequently imaged using sPA. The lesions identified by PA imaging are shown in Fig. 6(c) and were confirmed by gross pathology in Fig. 6(d). The measured lesion dimensions for all three samples are summarized in Table 1. The results show strong agreement between PA and gross pathology measurements, demonstrating that the sPA imaging accurately outlined lesion boundaries and dimensions.

**Table 1:** Lesion dimension measurement (W × D) by photoacoustic (PA) and gross pathology [unit: mm].

Sample	PA	Gross pathology	Absolute difference
1	9.77 × 9.59	9.44 × 9.18	0.33 × 0.41
2	9.23 × 10.16	9.89 × 10.97	0.66 × 0.81
3	11.72 × 6.71	12.75 × 6.85	1.03 × 0.14
Mean ± SD	—	—	0.67 ± 0.35 × 0.45 ± 0.34

### 4.3 *In Situ* Cadaver Ablation and Scanning

**Fig. 7:**
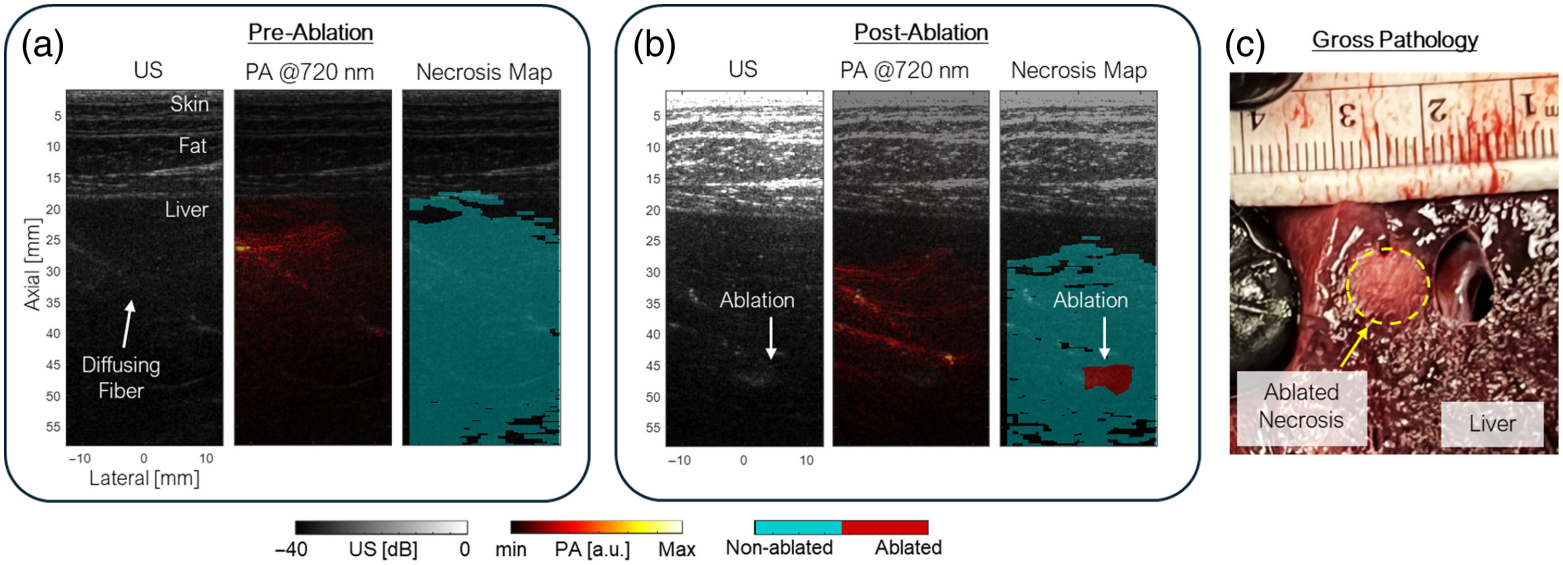
Experiment results: ultrasound image, photoacoustic (PA) image (720 nm) overlaid with US image and necrosis map (a) before and (b) after liver ablation. (c) Gross pathology post-procedure confirms the ablation-induced lesion necrosis.

The performance of sPA imaging using the diffusing fiber, combined with SHT-based artifact removal, was demonstrated *in situ* using a swine cadaver model for detecting ablation-induced lesions. Imaging was performed across wavelengths from 700 to 850 nm and processed using the delay-and-sum (DAS) beamforming algorithm.^39^ Figure 7 presents US/PA imaging results before [Fig. 7(a)] and after ablation [Fig. 7(b)]. Artifacts generated by the fiber were effectively suppressed via the SHT method, allowing clearer visualization of tissue signals. The fiber’s trajectory was visible in both imaging conditions. Necrosis mapping successfully revealed the ablation zone. Prior to ablation, images showed uniform blue labeling, whereas post-ablation results highlighted a circular ablated area. Lesion dimensions identified through PA imaging measured 7.81×6.31  mm, which closely matched post-procedural gross pathology findings, confirming a necrotic region with an average diameter of 7.93 mm.

**Fig. 8:**
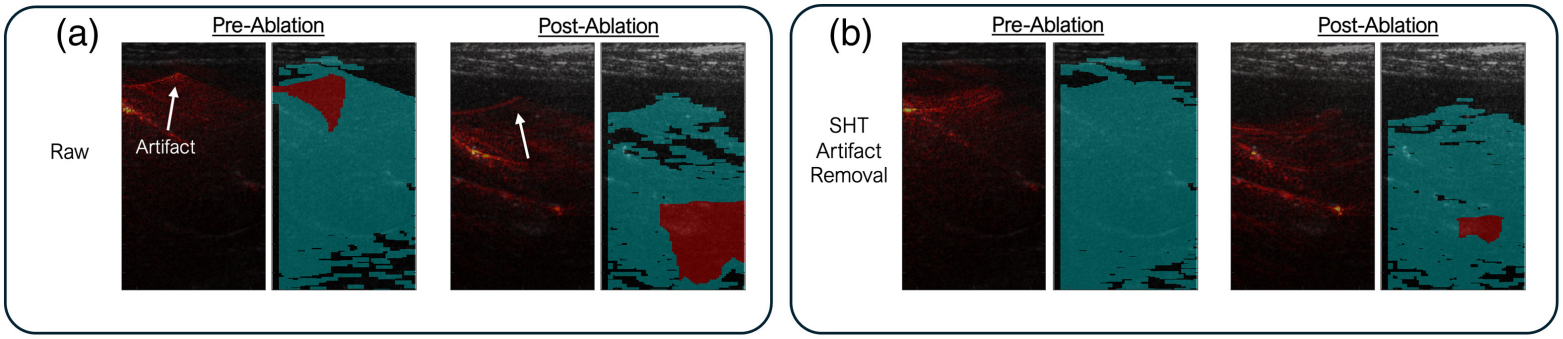
Comparison (a) before and (b) after the standard Hough transform (SHT) artifact removal.

Figure 8 illustrates necrosis maps generated before and after SHT correction. Without artifact removal [Fig. 8(a)], signal interference led to incorrect tissue labeling and overestimation of the ablation boundary. Following SHT application [Fig. 8(b)], labeling accuracy was restored, with the lesion correctly localized and delineated.

**Fig. 9:**
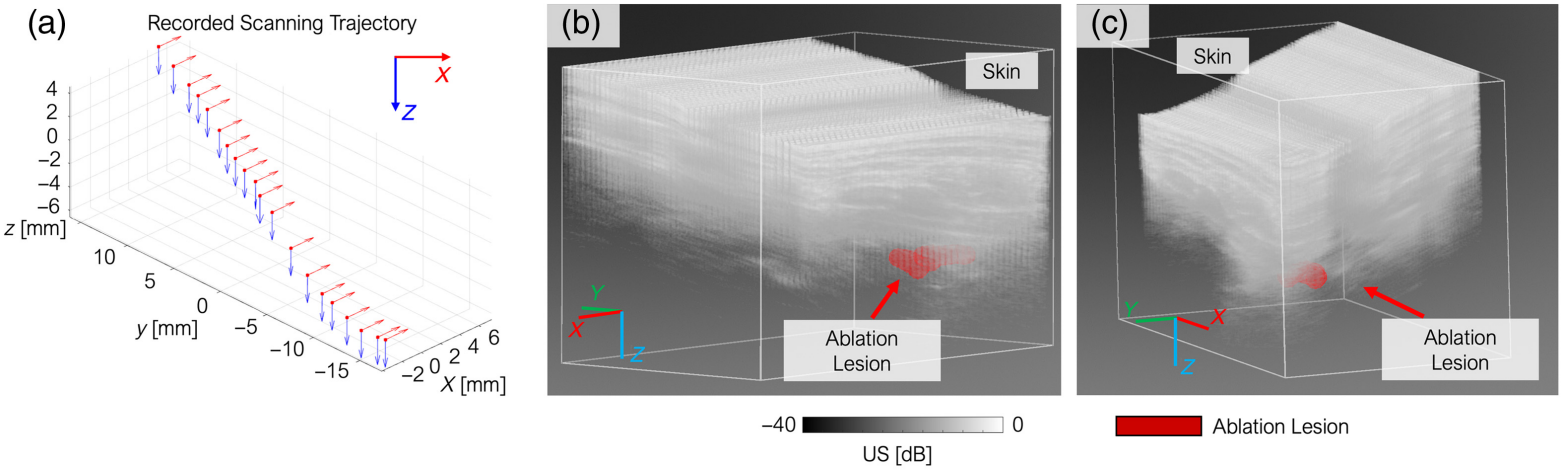
Reconstruction of post-ablation *in situ* liver lesion using robotic 3D scanning. (a) Trajectory of the ultrasound transducer along the abdominal skin surface recorded by the robotic system. (b), (c) 3D lesion maps reconstructed from photoacoustic imaging overlaid on anatomical structure, shown from two different viewing angles. Red voxels indicate the detected ablation-induced lesion, located in the shallow region of the liver beneath the skin.

The post-ablation area of the liver was scanned in 3D using a robotic system. A detailed 3D lesion map, along with an US image depicting the anatomical structure, was reconstructed based on the robot’s recorded scanning trajectory, as illustrated in Fig. 9. Figure 9(a) displays the path of the ultrasound transducer as it moved along the skin surface. The resulting 3D lesion mapping is presented in Figs. 9(b) and 9(c), viewed from two different angles for better spatial understanding.

The red voxels in the reconstructed image indicate the lesion site as identified by PA imaging. Notably, this lesion location aligns well with the anticipated ablation zone within the superficial region of the liver, just beneath the abdominal skin layer. The lesion volume was measured in three dimensions, yielding dimensions of 7.55×5.37×7.42  mm along the robot base frame coordinates. This spatial characterization provides critical validation of both the imaging system and the precision of the ablation procedure.

## 5 Discussion

This study demonstrates a multifaceted framework for minimally invasive and accurate monitoring of RFA-induced necrosis in the liver, leveraging a diffusing fiber-based PA imaging system, advanced artifact removal techniques, and robotic 3D lesion mapping. Collectively, the findings highlight the clinical potential of integrating PA imaging into conventional US-guided liver ablation workflows to improve real-time visualization and confirmation of treatment efficacy.

The use of a diffusing optical fiber for PA illumination provided a significant improvement in imaging homogeneity compared with traditional tip-illumination methods. Target scanning experiments demonstrated that the diffusing fiber enabled a more uniform light distribution, reducing PA signal disparity between centerline and peripheral targets. This consistent excitation field allowed for enhanced lesion mapping accuracy and spatial reliability, addressing a longstanding challenge in PA imaging for ablation monitoring. In our previous study,^23^ we demonstrated the use of sPA imaging to quantify ablation-induced lesions with submillimeter precision and strong agreement with histopathological measurements. The current work builds upon that foundation by integrating robotic control and diffusing fiber illumination to extend the technique toward large-area, intraoperative applications. The *ex vivo* validation further supported the clinical feasibility of the approach, showing that sPA imaging effectively delineated the ablation boundary. The mapped lesion sizes closely corresponded with gross pathological measurements, suggesting that diffusing fiber-based PA imaging could offer a reliable, real-time tool for monitoring and confirming complete ablation, potentially reducing recurrence rates in clinical practice.

Complementing the hardware innovation, this work introduced an artifact suppression framework based on the SHT method, which effectively masked channel signal sidelobe interference. This enhancement led to significant improvements in image clarity post-beamforming and enabled accurate spectral analysis. The preserved spectral integrity of the PA signal, even when acquired from the skin surface through multilayer tissue, demonstrated the robustness of the imaging setup in a clinically relevant configuration. Importantly, the fiber alignment and insertion did not disrupt the standard US-guided workflow, maintaining compatibility with existing ablation procedures.

3D lesion mapping using a robot-actuated PA-US scanning platform was successfully demonstrated in this work. By tracking the trajectory of the US transducer across the abdominal surface, the robotic system reconstructed high-resolution, volumetric lesion maps. Red voxels in the 3D renderings accurately marked the necrotic region, corroborating the expected ablation zone in the superficial liver area. This integration of robotic scanning with PA imaging adds an essential dimension of spatial awareness, offering post-ablation assessment intraoperatively. The quantified lesion dimensions (7.55×5.37×7.42  mm) show consistency with the gross-pathology measurement at 2D slice, further verify the precision and effectiveness of the ablation under guided imaging.

Despite the promising results, several limitations must be addressed for successful clinical translation. First, the current evaluations were performed in a cadaveric swine model, with the ablation confined to the superficial region of the liver. In clinical scenarios, liver tumors are often located deeper and can exceed several centimeters in diameter. The imaging system, including both illumination and acoustic detection, must be further optimized for such deeper and larger targets. Specifically, the maximum effective illumination volume of the diffusing fiber has not been fully characterized. Future studies should investigate fiber designs capable of illuminating larger regions, potentially through increased diffusion lengths or multifiber configurations. Moreover, deeper tissue imaging would benefit from a phased array US transducer with a lower center frequency to enhance acoustic penetration and increase the field of view (FOV). Current experiments did not address these requirements, which will be crucial for broader applicability.

In addition, we noted asymmetry in the PA intensity distribution, with higher signal levels in deeper tissue. This may result from the beamforming aperture or acoustic clutter near the fiber. These issues should be addressed through refined illumination strategies and beamforming algorithms to ensure consistent imaging across depth ranges. Another limitation is the lack of real-time integration between the robotic system and imaging feedback. The current robotic scanning was performed in an open-loop manner without using real-time US or PA guidance for motion control. Incorporating visual servoing^40^ and adaptive path planning techniques using real-time lesion detection in the image space will be critical for advancing toward autonomous or semi-autonomous robotic ablation systems. In addition, the system currently lacks motion compensation for respiration or other physiological movements, which must be accounted for in future *in vivo* trials. The chemical and mechanical safety of the etched diffusing fiber also remains unverified. For clinical deployment, the biocompatibility of the fiber materials and the structural integrity during insertion and manipulation must be thoroughly evaluated.

## 6 Conclusion

In this work, we presented a robot-assisted PA imaging system for intraoperative assessment of liver RFA, highlighting several key advancements. The integration of a diffusing optical fiber enabled broad, uniform illumination from a single insertion point, significantly enhancing lesion boundary visibility compared with traditional tip-illumination methods. Combined with spectroscopic analysis, SHT-based artifact suppression, and 3D robotic scanning, our system demonstrated accurate, volumetric lesion mapping *in situ* on a swine cadaver model, closely matching gross pathology findings. These results underscore the system’s potential to improve real-time ablation monitoring and reduce tumor recurrence. Future work will focus on optimizing light delivery and acoustic detection for deeper and larger lesions, enhancing real-time robotic feedback and motion compensation, and validating the safety and biocompatibility of the diffusing fiber to enable *in vivo* clinical translation.

## Data Availability

The data that support the findings during the current study are available from the corresponding author upon reasonable request.
